# EGFR-targeted fluorescent imaging using the da Vinci® Firefly™ camera for gallbladder cancer

**DOI:** 10.1186/s12957-022-02675-2

**Published:** 2022-06-15

**Authors:** Jung Ha Choi, Chang Moo Kang, Jeong Youp Park

**Affiliations:** 1 Division of Gastroenterology, Department of Internal Medicine, Yonsei University College of Medicine, Seoul, South Korea; 2 Division of HBP Surgery, Department of Surgery, Yonsei University College of Medicine, Seoul, South Korea; 3 Pancreatobiliary Cancer Center, Yonsei Cancer Center, and Yonsei Institute of Gastroenterology, Severance Hospital, Seoul, South Korea

**Keywords:** Gallbladder cancer, EGFR, Imaging, Robotic surgery

## Abstract

**Background:**

Fluorescent imaging may aid with the precise diagnosis and treatment of patients with gallbladder cancer. In this study, we sought to demonstrate whether the da Vinci**®** surgical system and Firefly™ camera could detect EGFR-targeted fluorescent images in orthotopic mouse models of gallbladder cancer.

**Methods:**

An orthotopic mouse model of gallbladder cancer was created by injecting NOZ gallbladder cancer cells mixed with Matrigel into the gallbladder. In vivo imaging of subcutaneous and orthotopic gallbladder tumors was performed after the injection of DyLight 650- or 800-conjugated EGFR antibody.

**Results:**

Western blotting, flow cytometry, and confocal microscopy showed the presence of EGFR in NOZ cells, but not in HEK293 cells. Subcutaneous NOZ cell tumors fluoresced after injection with fluorescent EGFR antibody, but subcutaneous HEK293 tumors did not. Fluorescent EGFR antibody made orthotopic NOZ tumors fluoresce, with an intensity stronger than that in the surrounding normal tissues. Histochemical examination confirmed the location of the tumors inside the gallbladder and adjacent liver parenchyma. Fluorescent signal was also detected in orthotopic gallbladder tumors with Firefly™ camera.

**Conclusion:**

Our study showed that fluorescent EGFR antibodies and the Firefly camera in the da Vinci system can detect fluorescing gallbladder tumors, which demonstrates their potential use for molecular imaging-based prevision surgery in the near future.

## Background

The incidence of gallbladder cancer worldwide is 2.2 individuals per 100,000 and 2.9 individuals per 100,000 in South Korea [1–3]. In advanced stage of the disease, mortality rate is high, with a 5-year survival rate of only 2.5%. However, when the tumor is confined to the gallbladder, the survival rate is 73.5% [3]. Thus, early detection and treatment are important for survival. However, distinguishing malignant disease from benign inflammatory disease is difficult and often makes a diagnosis tricky. The diagnosis of gallbladder cancer after cholecystectomy often occurs incidentally, as the presence of cancer is often not suspected [4].

Preclinical and clinical studies showed that when combined with endoscopy, laparoscopy and robotic surgery system fluorescent imaging aid in the detection of cancer in various organs and allow complete resection of the cancer [5, 6]. Fluorescent imaging may also help in the precise diagnosis and treatment of patients with gallbladder cancer [5, 7]. The Firefly™ camera, which can be integrated into the da Vinci® system, is a fluorescent imaging system that is currently used in clinical settings and can detect both visible and near-infrared light images. Its current applications include fluorescent cholangiography, bowel perfusion assessment, lymph node mapping, and sentinel lymph node biopsy [8]. To further expand the use of fluorescent imaging technology to tumor detection and focus surgical resection, molecular imaging methods with several contrast agents targeting various cancer biomarkers are currently under development [9]. Since laparoscopic and robotic surgeries are already being widely used for gallbladder surgery and suitable imaging tools are available, a fluorescent molecular imaging technology can be easily applied to treat gallbladder cancer after its usefulness is proven in preclinical studies [10–12].

The epidermal growth factor receptor (EGFR) is one of the most frequently examined cancer biomarkers in fluorescent imaging, as it is frequently altered in various human cancers [13]. In normal tissues, EGFR is a regulator of epithelial tissue development and homeostasis, but in cancerous tissues, EGFR is associated with cell proliferation, migration and metastases, evasion from apoptosis, and angiogenesis [14]. In gallbladder cancer, EGFR is expressed in about 90% of cases, making it an attractive target for use in fluorescent imaging [15, 16].

In this study, we sought to demonstrate that the da Vinci Firefly camera and EGFR-targeted antibody conjugated with fluorescent material could be used for the detection of gallbladder cancer in tumor mouse models.

## Materials and methods

### Gallbladder cancer cell lines

The human gallbladder cancer cell line, NOZ (EGFR-positive), and the human embryonic kidney cell lines, HEK293 (EGFR-negative) [17, 18], were maintained in RPMI 1640 and Minimum Essential Medium containing Earle’s balanced salts (MEM/EBSS) medium supplemented with 10% fetal bovine serum (Gibco, Carlsbad, USA) and an antibiotic-antimycotic solution (Gibco, Carlsbad, USA). All cells were cultured at 37°C in a 5% CO_2_ incubator.

### Mice

BALB/c nude mice (Central Lab Animal, Inc., Seoul, Korea), 4–5 weeks old, were used in the study. The mice were kept in a barrier facility under HEPA filtration and fed with an autoclaved laboratory rodent diet. All surgical and imaging procedures of the mice were performed after anesthetization by an intramuscular injection of 60% tiletamine and zolazepam and 40% xylazine HCl (0.02 ml) for anesthesia. Animals received antibiotics immediately prior to surgery and once per day for 3 days. The condition of the animals was monitored every day. CO_2_ inhalation was used for euthanasia.

### Antibody-dye conjugation

Mouse monoclonal antibodies to EGFR (199.12; Thermo Scientific, Rockford, IL, USA) were conjugated to DyLight 650 or DyLight 800 dyes (Thermo Scientific, Rockford, IL, USA) after removing the bovine serum albumin (BSA) using Pierce™ Antibody Clean-up Kit (Thermo Scientific, Rockford, IL, USA) according to the manufacturer’s specifications.

### Western blotting

The cell were lysed in lysis buffer containing 70 mM β-glycerophosphate, 0.6 mM sodium orthovanadate, 2 mM MgCl2, 1 mM ethylene glycol tetraacetic acid, 1 mM DTT (Invitrogen, Grand Island, NY, USA), 0.5% Triton-X100, 0.2 mM phenylmethylsulfonyl fluoride, and 1% protease inhibitor cocktail (SigmaAldrich, St. Louis, MO, USA). The lysates were separated via sodium dodecylsulfate–polyacrylamide gel electrophoresis (SDS-PAGE) and transferred to polyvinylidene fluoride membranes (Millipore, Billerica, MA, USA). The membranes were blocked in 5% (w/v) non-fat dry milk and probed with anti-EGFR (D-8, Santa Cruz, Dallas, TX, USA) at a dilution of 1:100. The immunoreactive proteins were visualized using the SuperSignal West Pico Chemiluminescent Substrate (Thermo Scientific).

### Flow cytometry and confocal microscopy

A total of 2 × 10^6^ cells were washed with phosphate-buffered saline (PBS) and fixed in 4% paraformaldehyde for 15 min at room temperature in the dark. The cells were washed and incubated with 1% BSA for 1 h at room temperature. After incubation, the cells were washed with PBS and incubated with 0.5 nM anti-EGFR (199.125; Thermo Scientific, Rockford, IL, USA) for 3 h at room temperature. The cells were then incubated with Alexa fluor 488-conjugated polyclonal goat anti-mouse secondary antibody (Jackson ImmunoResearch Laboratories, Inc., USA) for 1 h at room temperature. After washing with PBS, fluorescence was detected with BD FACS LSRII SORP system (Becton Dickinson Company, New Jersey, USA), and confocal microscopy was performed using an LSM 700 instrument (Carl Zeiss, Oberkochen, Germany).

### Subcutaneous and orthotopic mouse models of gallbladder cancer

To make the subcutaneous mouse models of gallbladder cancers, NOZ and HEK293 cells (2 × 10^6^ cells) were injected subcutaneously into the flanks of BALB/c nude mice. When the size of the subcutaneous tumors grew to 10–20 mm in diameter, imaging was performed. To create the orthotopic mouse models of gallbladder cancer, 2 × 10^6^ NOZ cells were mixed with Matrigel (Corning, Arizona, USA). The gallbladders were exposed and injected with cell suspension using a 29-G insulin syringe (BD, USA). The abdominal walls were closed using Vicryl 6/0. Imaging was performed 5 weeks after the injection [19].

### Fluorescent immunostaining

Formalin-fixed, paraffin-embedded tissue samples were sectioned into serial 4-mm slices and placed on microscope slides. After deparaffinization in xylene and rehydration in alcohol, tissue sections underwent antigen retrieval for 30 min in Tris-EDTA buffer titrated to pH 9.0 at 97°C and cooled with tap water. Non-specific antigen reactions were blocked by a 1-h incubation with M.O.M. mouse IgG blocking buffer. The slides were then incubated with the anti-EGFR (MA5-13319; Thermo Scientific, Rockford, IL, USA) at a dilution of 1:500 for 12–16 h at 4°C. The slides were then incubated with Alexa fluor 488-conjugated polyclonal goat anti-mouse secondary antibody (Jackson ImmunoResearch Laboratories, Inc., USA) at a dilution of 1:100 for 1 h at room temperature. After washing with PBS, the slides were examined under confocal microscopy using LSM 700 instrument (Carl Zeiss, Oberkochen, Germany).

### Imaging

Mice harboring subcutaneous and orthotopic tumors were injected with anti-EGFR (199.12; Thermo Scientific, Rockford, IL, USA) conjugated to DyLight 650 via the tail vein. Imaging was then performed using an IVIS®Spectrum In Vivo Imaging System (Perkin Elmer, Waltham MA, USA). For subcutaneous tumor, imaging was done on days 1, 2, and 3 after injection under anesthesia. For orthotopic tumor in each mouse, imaging was done after euthanasia, opening of the abdominal wall, and exposure of the liver and gallbladder. After the in vivo imaging of orthotopic tumors, the tumors and livers were removed from the mice, and ex vivo imaging was performed. The Firefly™ camera (Intuitive Surgical Inc., Sunnyvale, CA, USA) integrated into the da Vinci® robotic surgical system (Intuitive Surgical Inc., Sunnyvale, CA, USA) was used to image and record the fluorescence of anti-EGFR (199.12; Thermo Scientific, Rockford, IL, USA) conjugated to DyLight 800 in orthotopic tumors. The tumor background ratio was analyzed by ImageJ (National Institutes of Health, Bethesda, Maryland). The tumor was resected after in vivo florescent imaging. Subsequently, hematoxylin and eosin staining of resected orthotopic tumors was performed, and the samples were observed under light microscopy.

## Results

### Expression of EGFR in gallbladder cancer cells

**Fig. 1:**
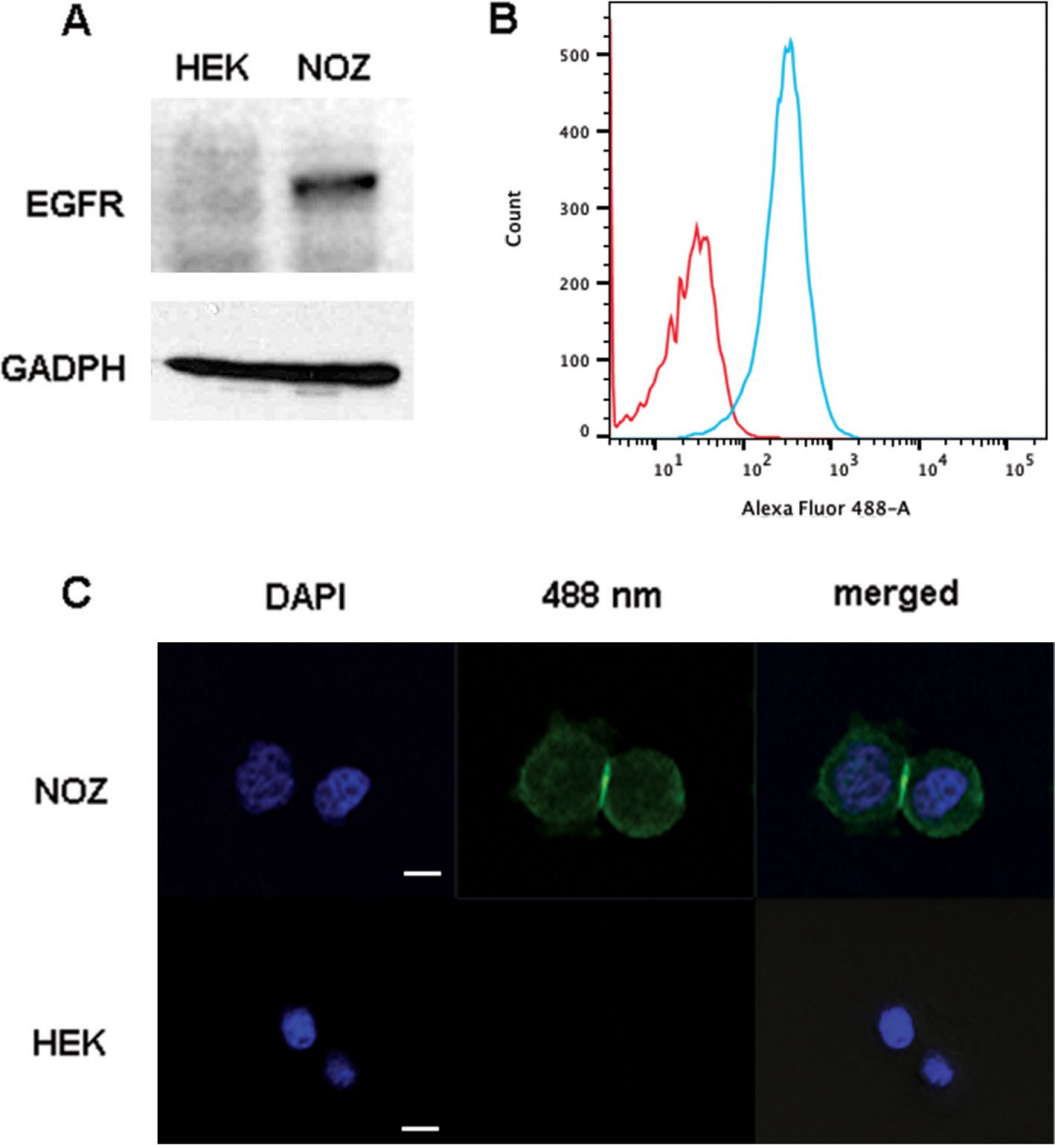
Characterization of gallbladder tumor cell lines. A Western blot analysis of EGFR expression in NOZ cells (right lane) and HEK293 cells (left lane). B Flow cytometric analysis of fluorescent anti-EGFR bounding in NOZ cells (blue line) and HEK293 cells (red line). C Labeling of NOZ and HEK293 cells with fluorescent anti-EGFR (×40 water immersion objective with the LSM 700 confocal microscope, using 405- and 488-nm lasers). The white scale bar represents 10 μm

Western blotting, flow cytometry, and confocal microscopy were performed to confirm that EGFR was expressed in gallbladder cancer cells. Fluorescent anti-EGFR was able to bind to the membranes of cancer cells. Western blotting was used to detect the expression of EGFR in NOZ cells, but not in HEK293 cells (Fig. 1A). Likewise, flow cytometry was used to show that anti-EGFR bound to NOZ cells, but not to HEK293 cells (Fig. 1B). Confocal microscopy was also used to show the presence of fluorescence foci along the membranes of NOZ cells, but not HEK293 cells, indicating that the fluorescent anti-EGFR bound to the cell membranes of EFGR-positive NOZ cells (Fig. 1C).

### Imaging subcutaneous tumors with fluorescent anti-EGFR

**Fig. 2:**
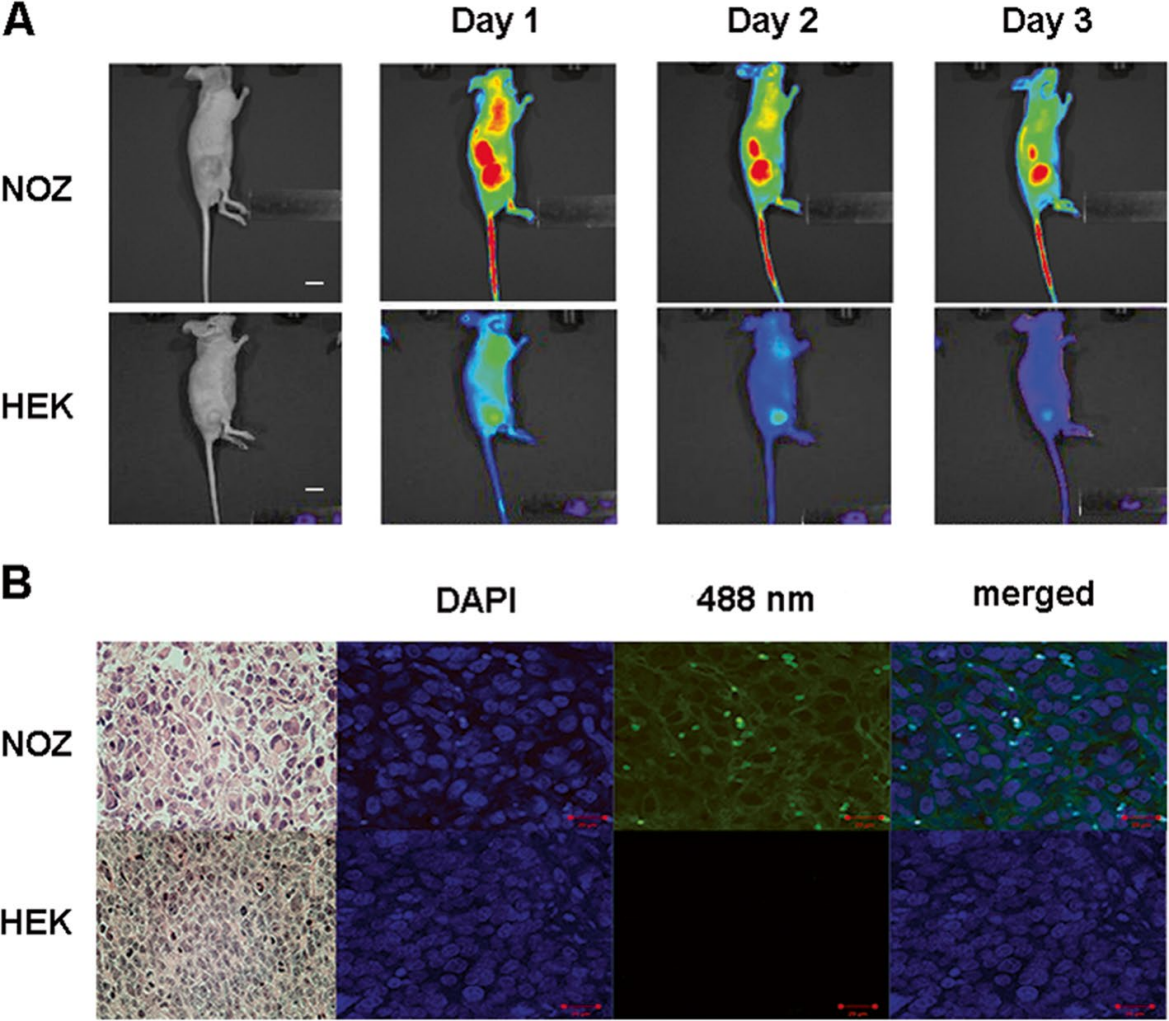
Imaging of EGFR in subcutaneously-transplanted gallbladder tumors in vivo. **A** Representative images of NOZ and HEK293 cell tumors. The mouse was imaged under both white light and fluorescence illumination (IVIS, using a 650-nm laser). **B** NOZ and HEK293 cell tumors stained hematoxylin and eosin staining (×200) and fluorescent anti-EGFR (×200, using 405- and 488-nm lasers). The red scale bar represents 100 μm

Subcutaneous tumors were grown for about 4 weeks after injecting the cells in the flanks of mice. Dylight 650-conjugated anti-EGFR was injected into the tail veins of the mice, and then, the tumors were imaged. Twenty-four hours after injection, a strong fluorescent signal was detected in EGFR-positive NOZ cell tumors, but the signal was faint in EGFR-negative HEK293 cell tumors. Over time, the intensity of the fluorescent signal decreased, persisting up to 72 h in the NOZ cell tumors, and while almost disappearance in the HEK293 cell tumors (Fig. 2A).

Analysis of the immunofluorescence from the anti-EGFR in resected subcutaneous tumors from the mice confirmed the expression of EGFR in NOZ cell tumors, but not in HEK293 cells tumors (Fig. 2B).

### Imaging orthotopic tumors with fluorescent EGFR antibodies

**Fig. 3:**
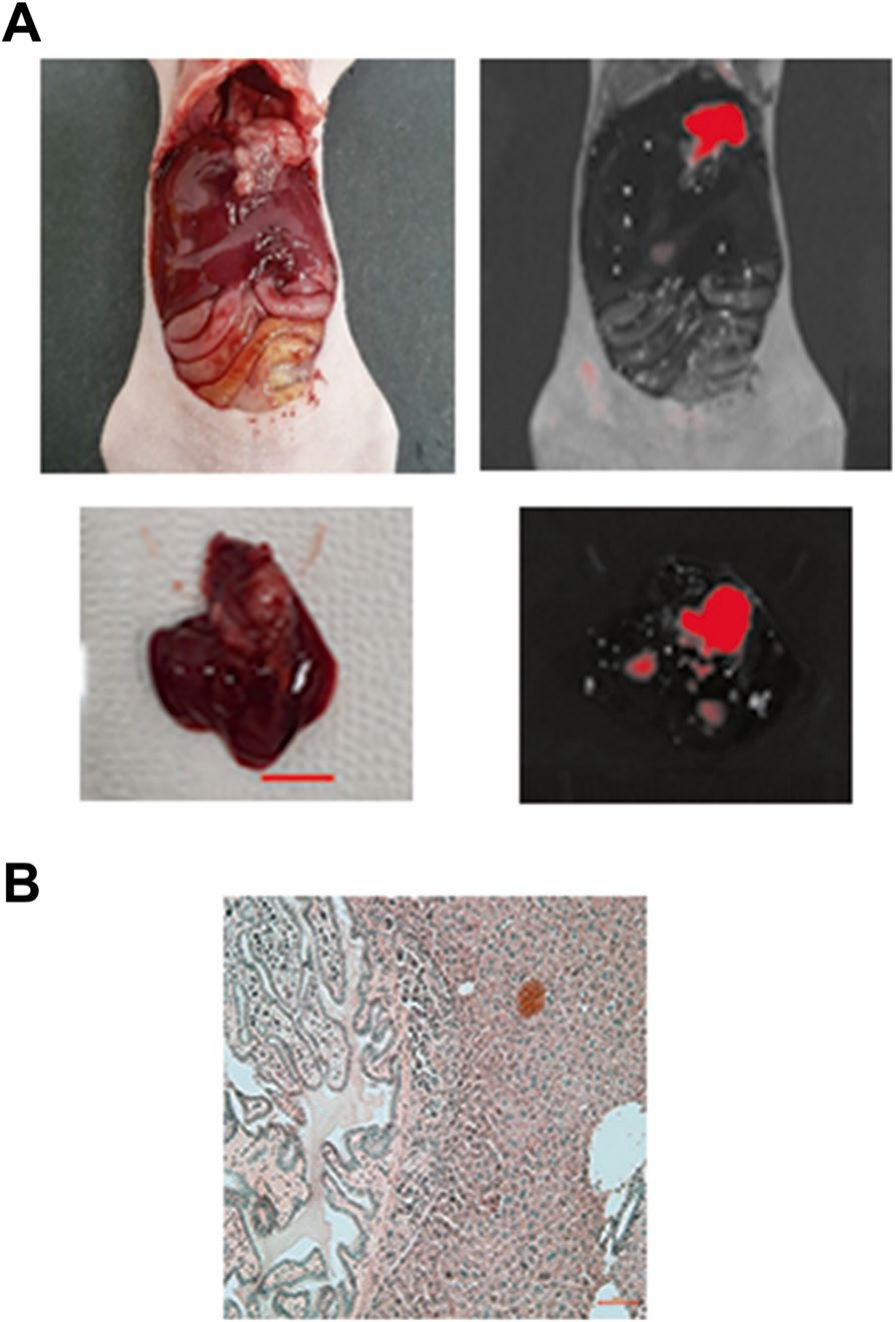
Imaging of EGFR in orthotopically transplanted NOZ cell gallbladder tumors with IVIS. **A** Fluorescence signals from orthotopically transplanted gallbladder tumors (using a 650-nm laser). The red scale bar represents 1 cm. **B** Hematoxylin and eosin staining of orthotopically transplanted NOZ cell gallbladder tumors and normal liver tissues (×200). The red scale bar represents 100 μm

NOZ cells mixed with Matrigel were injected into gallbladders. After 5 weeks, Dylight 650-conjugated anti-EGFR was injected into the tail veins of the mice. Twenty-four hours after injecting the antibody, each mouse was sacrificed and the abdomen was opened. Upon gross examination, tumors were noted in the gallbladders of the mice. Fluorescent imaging detected strong intense signals from the tumors, which was lacking in surrounding normal tissues. Ex vivo imaging confirmed the fluorescence came from the tumors (Fig. 3). No fluorescent signal was noted in the absence of tumors (data not shown). When the tumor was present and absent, the tumor-to-background ratios of fluorescence signal were 2.61 and 1.19, respectively. Subsequent histologic examination confirmed tumor in the gallbladder and adjacent liver parenchyma (Fig. 3). When no fluorescence was detected, histologic examination only found inflammatory cells in the gallbladder and liver (data not shown).

### Detection of fluorescence from orthotopic tumors using firefly camera and da Vinci system

**Fig. 4:**
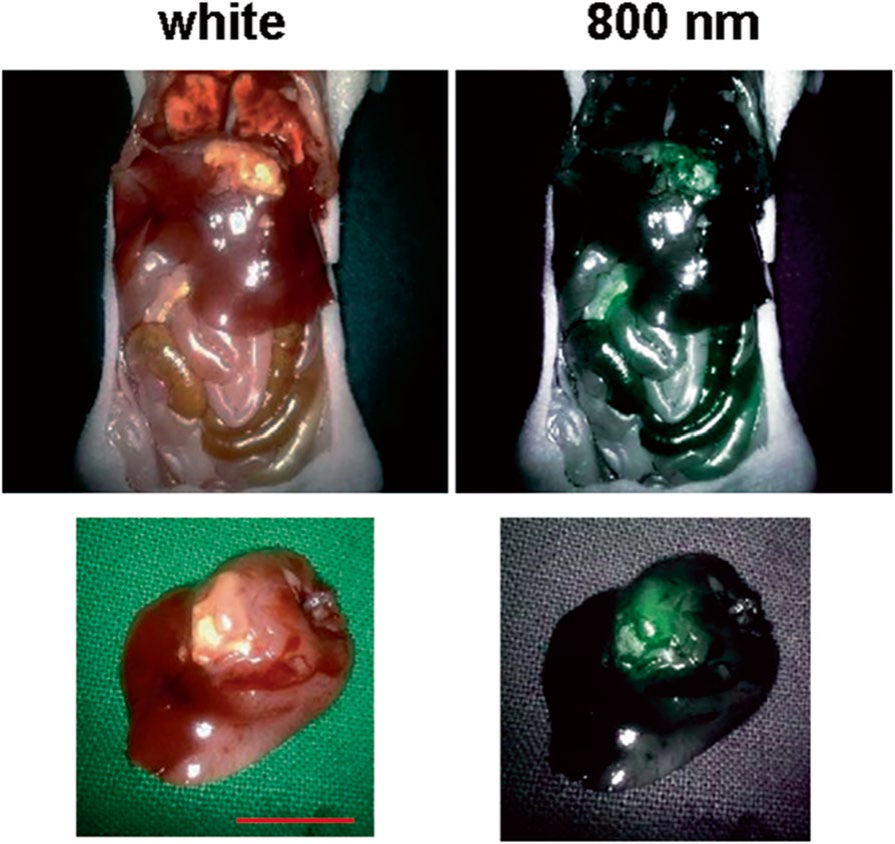
Imaging of EGFR in orthotopically transplanted NOZ gallbladder tumors using the Firefly camera. Fluorescence signals from gallbladder tumors orthotopically transplanted in the gallbladder were detected (using an 800-nm laser). The red scale bar represents 1 cm

The in vivo imaging system in our facility could detect 650 nm fluorescence, while the Firefly camera could detect only 800 nm fluorescence. Therefore, imaging orthotopic tumors using the Firefly camera was done with Dylight 800-conjugated anti-EGFR, as mentioned above. Strong fluorescent signals from the tumor detected with the Firefly™ camera could be easily seen on screen of robotic surgery system (Fig. 4). Some autofluorescence were also detected from the bowels. Ex vivo imaging also confirmed that the fluorescence came from the tumor (Fig. 4).

## Discussion

Our results showed that anti-EGFR specifically bound to gallbladder tumors and produced fluorescence, which could be detected by imaging during surgery in real time. In vitro and in vivo fluorescent EGFR antibody could make EGFR-positive gallbladder tumor fluorescent; however, when no tumor was made or EGFR was not expressed, no fluorescence was emitted. In addition, using 800-nm fluorescent EGFR antibody Firefly™ camera in da Vinci® robotic surgery system could detect fluorescent signal from gallbladder tumor, suggesting that its use is not limited to robotic surgery, but can also be expanded to laparoscopic and endoscopic system.

The results of the present study implicated that fluorescent imaging technology could contribute to the differentiation of malignant gallbladder tumor from benign inflammatory disease, which could help in avoiding incomplete resection of incidental gallbladder cancer or misdiagnosis of gallbladder tumor. In this study, we did not use cholecystitis animal model for comparison; however, when no tumor was made in orthotopic tumor models, no fluorescent signal was detected and histologically only signs of inflammation was noted. Furthermore, assessing tumor margin and lymph node metastasis might also help in achieving complete resection [7]. Pathological R0 resection are usually determined by final pathological examination after radical cholecystectomy. However, clinical R0 can be assumed, if fluorescent imaging can determine margin-negative simple cholecystectomy with no evidence of regional lymph node metastasis or grossly residual cancer tissues during surgery [20, 21]. Since simple cholecystectomy is regarded as feasible and safe in the early stage of gallbladder cancer [22–24], more appropriate selection criteria of simple cholecystectomy could be established and major surgery can be avoided, based on the present EGFR-targeted florescent imaging.

EGFR-targeted fluorescent imaging has been tested in clinical trials for several types of cancer, including head and neck cancer [25], pancreatic cancer [26], glioblastoma and glioma [13, 27], oral cancer [28], and lung cancer [13]. Fluorescence-guided surgical imaging using panitumumab, an EGFR antibody, conjugated to IRDye800 was used to detect gliomas in real time [29], demonstrating good tumor-to-background contrast ratio. The same antibody was used in a murine model of colorectal cancer for fluorescence-guided surgery with a strong fluorescent contrast [30]. Additionally, the gallbladder is a good candidate for fluorescent imaging using EGFR-targeted antibodies, since EGFR is overexpressed in gallbladder cancer regardless of the cancer stage [31]. Our in vitro test confirmed that NOZ (gallbladder cancer cell line) and other bile duct cancer cell lines, such as SNU308 (gallbladder cancer), SNU478 (ampulla of vater cancer), and SNU1196 (hilar cancer), also expressed EGFR (data not shown). How high the EGFR expression should be for successful fluorescent imaging would be a suitable topic for future research. Other than EGFR, folate receptor [32], vascular endothelial growth factor [33], carcinoembryonic antigen [34], cathepsins [35], and matrix metalloproteinase [36] have been tested as targets for fluorescent imaging. Additional studies are needed to find other suitable biomarkers for fluorescent imaging of gallbladder cancer.

Various orthotopic tumor models have been used to develop new laparoscopic and robotic surgical techniques, as they mimic the anatomical characteristics of tumors [37]. Considering the current status of minimally invasive radical cholecystectomy in gallbladder cancer, the present study further emphasizes the usefulness of orthotopic tumor models in testing new surgical techniques for gallbladder cancer. In the near future, more precise decision-making on surgical extent according to florescent molecular images would be tested using the da Vinci surgical system in orthotopic tumor models of gallbladder cancer.

## Conclusion

In conclusion, our study showed that a fluorescent EGFR-targeted antibody can be used successfully for the diagnosis of gallbladder tumors, which in turn can help detect malignant disease. The use of Firefly™ camera in the da Vinci**®** robotic system allowed the detection of gallbladder tumors via fluorescence, which demonstrated the potential of molecular imaging using this robotic surgical system. Further studies are needed for the clinical application of molecular imaging using fluorescence technology.

## Data Availability

Not applicable.

## Abbreviation

EGFR: Epidermal growth factor receptor

## References

[1] Ferlay J, Soerjomataram I, Dikshit R, Eser S, Mathers C, Rebelo M (2015). Cancer incidence and mortality worldwide: sources, methods and major patterns in GLOBOCAN 2012. Int J Cancer.

[2] Kim BW, Oh CM, Choi HY, Park JW, Cho H, Ki M (2019). Incidence and overall survival of biliary tract cancers in South Korea from 2006 to 2015: using the National Health Information Database. Gut Liver.

[3] Wi Y, Woo H, Won YJ, Jang JY, Shin A (2018). Trends in gallbladder cancer incidence and survival in Korea. Cancer Res Treat.

[4] Hickman L, Contreras C (2019). Gallbladder cancer: diagnosis, surgical management, and adjuvant therapies. Surg Clin North Am.

[5] Hiroshima Y, Maawy A, Metildi CA, Zhang Y, Uehara F, Miwa S (2014). Successful fluorescence-guided surgery on human colon cancer patient-derived orthotopic xenograft mouse models using a fluorophore-conjugated anti-CEA antibody and a portable imaging system. J Laparoendosc Adv Surg Tech A.

[6] van der Vorst JR, Schaafsma BE, Hutteman M, Verbeek FP, Liefers GJ, Hartgrink HH (2013). Near-infrared fluorescence-guided resection of colorectal liver metastases. Cancer..

[7] Ahmad A (2020). Use of indocyanine green (ICG) augmented near-infrared fluorescence imaging in robotic radical resection of gallbladder adenocarcinomas. Surg Endosc.

[8] Dip FD, Ishizawa T, Kokudo N, Rosenthal RJ (2015). Fluorescence imaging for surgeons : concepts and applications.

[9] Wendler T, van Leeuwen FWB, Navab N, van Oosterom MN (2021). How molecular imaging will enable robotic precision surgery: the role of artificial intelligence, augmented reality, and navigation. Eur J Nucl Med Mol Imaging.

[10] Yoon YS, Han HS, Cho JY, Choi Y, Lee W, Jang JY (2015). Is laparoscopy contraindicated for gallbladder cancer? A 10-year prospective cohort study. J Am Coll Surg.

[11] Shirobe T, Maruyama S (2015). Laparoscopic radical cholecystectomy with lymph node dissection for gallbladder carcinoma. Surg Endosc.

[12] Shenoy R, Mederos MA, Ye L, Mak SS, Begashaw MM, Booth MS (2021). Intraoperative and postoperative outcomes of robot-assisted cholecystectomy: a systematic review. Syst Rev.

[13] Hernot S, van Manen L, Debie P, Mieog JSD, Vahrmeijer AL (2019). Latest developments in molecular tracers for fluorescence image-guided cancer surgery. Lancet Oncol.

[14] Sigismund S, Avanzato D, Lanzetti L (2018). Emerging functions of the EGFR in cancer. Mol Oncol.

[15] Das C, Mukhopadhyay M, Subba S, Saha AK, Mukhopadhyay B (2021). Role of EGFR and HER-2/NEU expression in gall bladder carcinoma (GBC). J Lab Physicians.

[16] Kaufman M, Mehrotra B, Limaye S, White S, Fuchs A, Lebowicz Y (2008). EGFR expression in gallbladder carcinoma in North America. Int J Med Sci.

[17] Lill NL, Douillard P, Awwad RA, Ota S, Lupher ML, Miyake S (2000). The evolutionarily conserved N-terminal region of Cbl is sufficient to enhance down-regulation of the epidermal growth factor receptor. J Biol Chem.

[18] Kikuchi O, Ohashi S, Horibe T, Kohno M, Nakai Y, Miyamoto S (2016). Novel EGFR-targeted strategy with hybrid peptide against oesophageal squamous cell carcinoma. Sci Rep.

[19] Mita Y, Ajiki T, Kamigaki T, Okazaki T, Hori H, Horiuchi H (2007). Antitumor effect of gemcitabine on orthotopically inoculated human gallbladder cancer cells in nude mice. Ann Surg Oncol.

[20] Kang CM, Choi GH, Park SH, Kim KS, Choi JS, Lee WJ (2007). Laparoscopic cholecystectomy only could be an appropriate treatment for selected clinical R0 gallbladder carcinoma. Surg Endosc.

[21] Kang CM, Lee WJ, Choi GH, Kim JY, Kim KS, Choi JS (2007). Does "clinical" R0 have validity in the choice of simple cholecystectomy for gallbladder carcinoma?. J Gastrointest Surg.

[22] Lee H, Kwon W, Han Y, Kim JR, Kim SW, Jang JY (2018). Optimal extent of surgery for early gallbladder cancer with regard to long-term survival: a meta-analysis. J Hepatobiliary Pancreat Sci.

[23] Kang H, Choi YS, Suh SW, Choi G, Do JH, Oh HC (2021). Prognostic significance of tumor location in T2 gallbladder cancer: a systematic review and meta-analysis. J Clin Med..

[24] Lee SE, Kim KS, Kim WB, Kim IG, Nah YW, Ryu DH (2014). Practical guidelines for the surgical treatment of gallbladder cancer. J Korean Med Sci.

[25] Rosenthal EL, Warram JM, de Boer E, Chung TK, Korb ML, Brandwein-Gensler M (2015). Safety and tumor specificity of cetuximab-IRDye800 for surgical navigation in head and neck cancer. Clin Cancer Res.

[26] Tummers WS, Miller SE, Teraphongphom NT, Gomez A, Steinberg I, Huland DM (2018). Intraoperative pancreatic cancer detection using tumor-specific multimodality molecular imaging. Ann Surg Oncol.

[27] Miller SE, Tummers WS, Teraphongphom N, van den Berg NS, Hasan A, Ertsey RD (2018). First-in-human intraoperative near-infrared fluorescence imaging of glioblastoma using cetuximab-IRDye800. J Neuro-Oncol.

[28] van Keulen S, van den Berg NS, Nishio N, Birkeland A, Zhou Q, Lu G (2019). Rapid, non-invasive fluorescence margin assessment: optical specimen mapping in oral squamous cell carcinoma. Oral Oncol.

[29] Zhou Q, van den Berg NS, Rosenthal EL, Iv M, Zhang M, Vega Leonel JCM (2021). EGFR-targeted intraoperative fluorescence imaging detects high-grade glioma with panitumumab-IRDye800 in a phase 1 clinical trial. Theranostics..

[30] Marston JC, Kennedy GD, Lapi SE, Hartman YE, Richardson MT, Modi HM (2019). Panitumumab-IRDye800CW for fluorescence-guided surgical resection of colorectal cancer. J Surg Res.

[31] Doval DC, Azam S, Sinha R, Batra U, Mehta A (2014). Expression of epidermal growth factor receptor, p53, Bcl2, vascular endothelial growth factor, cyclooxygenase-2, cyclin D1, human epidermal receptor-2 and Ki-67: association with clinicopathological profiles and outcomes in gallbladder carcinoma. J Carcinog.

[32] Hoogstins CE, Tummers QR, Gaarenstroom KN, de Kroon CD, Trimbos JB, Bosse T (2016). A novel tumor-specific agent for intraoperative near-infrared fluorescence imaging: a translational study in healthy volunteers and patients with ovarian cancer. Clin Cancer Res.

[33] Harlaar NJ, Koller M, de Jongh SJ, van Leeuwen BL, Hemmer PH, Kruijff S (2016). Molecular fluorescence-guided surgery of peritoneal carcinomatosis of colorectal origin: a single-Centre feasibility study. Lancet Gastroenterol Hepatol.

[34] Boogerd LSF, Hoogstins CES, Schaap DP, Kusters M, Handgraaf HJM, van der Valk MJM (2018). Safety and effectiveness of SGM-101, a fluorescent antibody targeting carcinoembryonic antigen, for intraoperative detection of colorectal cancer: a dose-escalation pilot study. Lancet Gastroenterol Hepatol.

[35] Smith BL, Gadd MA, Lanahan CR, Rai U, Tang R, Rice-Stitt T (2018). Real-time, intraoperative detection of residual breast cancer in lumpectomy cavity walls using a novel cathepsin-activated fluorescent imaging system. Breast Cancer Res Treat.

[36] Unkart JT, Chen SL, Wapnir IL, Gonzalez JE, Harootunian A, Wallace AM (2017). Intraoperative tumor detection using a ratiometric activatable fluorescent peptide: a first-in-human phase 1 study. Ann Surg Oncol.

[37] Lee NP, Chan CM, Tung LN, Wang HK, Law S (2018). Tumor xenograft animal models for esophageal squamous cell carcinoma. J Biomed Sci.

